# To vaccinate or not to vaccinate? Perspectives on HPV vaccination among girls, boys, and parents in the Netherlands: a Q-methodological study

**DOI:** 10.1186/s12889-017-4879-2

**Published:** 2017-11-07

**Authors:** Nathalie J. S. Patty, Hanna Maria van Dijk, Iris Wallenburg, Roland Bal, Theo J. M. Helmerhorst, Job van Exel, Jane Murray Cramm

**Affiliations:** 1 Erasmus University Rotterdam, Erasmus School of Health Policy & Management, Burgemeester Oudlaan 50, 3062 PA Rotterdam, The Netherlands; 2 Erasmus MC, Department of Obstetrics & Gynaecology, Rotterdam, The Netherlands; 3 Erasmus University Rotterdam, Erasmus School of Economics, Rotterdam, The Netherlands

**Keywords:** Human papillomavirus, Vaccination, Perspective, Health belief model, Q-methodology, Boys, Girls, Parents

## Abstract

**Background:**

Despite the introduction of Human papillomavirus (HPV) vaccination in national immunization programs (NIPs), vaccination rates in most countries remain relatively low. An understanding of the reasons underlying decisions about whether to vaccinate is essential in order to promote wider spread of HPV vaccination. This is particularly important in relation to policies seeking to address shortfalls in current HPV campaigns. The aim of this study was to explore prevailing perspectives concerning HPV vaccination among girls, boys, and parents, and so to identify potential determinants of HPV vaccination decisions in these groups.

**Method:**

Perspectives were explored using Q-methodology. Forty-seven girls, 39 boys, and 107 parents in the Netherlands were asked to rank a comprehensive set of 35 statements, assembled based on the health belief model (HBM), according to their agreement with them. By-person factor analysis was used to identify common patterns in these rankings, which were interpreted as perspectives on HPV vaccination. These perspectives were further interpreted and described using data collected with interviews and open-ended questions.

**Results:**

The analysis revealed four perspectives: “prevention is better than cure,” “fear of unknown side effects,” “lack of information and awareness,” and “my body, my choice.” The first two perspectives and corresponding determinants of HPV vaccination decisions were coherent and distinct; the third and fourth perspectives were more ambiguous and, to some extent, incoherent, involving doubt and lack of awareness and information (perspective 3), and overconfidence (perspective 4).

**Conclusions:**

Given the aim of publically funded vaccination programs to minimize the spread of HPV infection and HPV-related disease and the concerns about current uptake levels, our results indicate that focus should be placed on increasing awareness and knowledge, in particular among those in a modifiable phase.

## Background

Human papillomavirus (HPV) is a common sexually transmitted disease in men and women. Most sexually active women and men become infected at some point in their lives, and infections may also reoccur [1]. Despite the transient nature of HPV, persistent infection with oncogenic HPV strains may cause cancer. Virtually 99% of cervical cancers in women are caused by genital HPV infection [2]. Worldwide, cervical cancer is the second most common cancer among women, resulting in 270,000 deaths annually [1]. Although cervical cancer has received much attention, oncogenic HPV strains also affect men [3, 4]. An estimated 90% of anal cancers, 60% of penile cancers, and 70% of oropharyngeal cancers are linked directly to oncogenic HPV strains [5]. In developed countries, the number of HPV-related (penile, oral, and anal) cancers in men has been estimated to be similar to that of cervical cancers in women [4], creating a high disease burden [3, 6].

In 2006, the European Medicines Agency and the US Federal Drug Administration approved the first vaccines against HPV. Because these were the first vaccines aimed at preventing cancer, wide policy debate about whether they should be included in national immunization programs (NIPs) ensued. The Netherlands, where the current study was conducted, was among the first countries to include the vaccine in its NIP, in 2008. However, this was only for girls and not for boys, which is currently still the case. Some countries that introduced the vaccine to their NIPs later also included HPV immunization for boys [7]. Despite the introduction of HPV vaccination in NIPs, vaccination rates in most countries remain relatively low. Countries that engage in school-based vaccination programs generally have significantly higher uptakes than do those that offer the vaccine in alternative schemes, and uptake is generally lower among boys than among girls in countries recommending immunization for both sexes [8].

As the rationale behind publically funded vaccination is to achieve high coverage and, thereby, herd immunity, understanding the reasons underlying the decisions of children and their parents about whether to vaccinate is important to achieve greater permeation of the practice. Such understanding is particularly important for policies aiming to address shortfalls in current HPV campaigns. Furthermore, the novelty of HPV vaccination, as the first immunization measure aimed at preventing cancer, serves as an interesting case providing insight into how this “new generation” of vaccines is valued among designated users. Therefore, the aims of this study were to explore prevailing perspectives on HPV vaccination among girls, boys, and parents, and to identify underlying determinants shaping decisions about whether to vaccinate against HPV.

### Determinants of vaccination against HPV

Much attention has been devoted to determinants influencing HPV vaccine uptake. Most studies have found that the *perceived benefits* of the vaccine have considerable effects on decisions about whether to vaccinate; the intention to vaccinate increases with the perceived benefits. Commonly reported benefits involve the perceived effectiveness of the vaccine [9–11] and beliefs that the vaccine will protect against or minimize the severity of HPV-related disease and/or promote the future health of recipients and their future partners and others in the community through herd immunity [9, 10, 12–14].

*Perceived barriers* also influence decisions about whether to vaccinate against HPV. Reported barriers entail the low perceived effectiveness of the vaccine, concerns about side effects and safety, fear of needles, fear of sexual disinhibition or promiscuity stigma, and lack of knowledge and awareness [9, 10, 14–18]. The latter involve misconceptions regarding the physical injection site [12] and the prophylactic effect of the vaccine [12, 19]. In addition, there is low awareness of the existence of HPV vaccination for men, as the vaccine has been advertised mainly for women. Thus, men have not adopted HPV immunization as a norm [20], potentially emasculating those who decide to be vaccinated [17]. Other barriers, such as lack of trust in the government (promoting the vaccine) and/or concerns about commercial interests of pharmaceutical companies, have also been reported to compromise HPV vaccine uptake [9, 10, 14, 15].

Another important determinant affecting decisions about whether to vaccinate is the *perceived severity* of HPV and perceived susceptibility to HPV-related diseases; most studies have shown that beliefs about the severity and likelihood of HPV infection and/or development of HPV-related diseases positively affect vaccination uptake [13–15, 20–22]. However, many girls perceive a low likelihood of contracting HPV, perhaps due to the young vaccination age [9, 10]. Studies have also suggested that anticipated regret of (in)action plays an important role in HPV vaccination decisions [12, 13, 23, 24]. Furthermore, general acceptance of other childhood vaccinations and professional endorsement have been shown to affect HPV vaccine acceptance [14, 15, 17, 25].

In addition to all of these beliefs, *demographic determinants* (e.g., education, religion), *social influences*, and *subjective norms* (e.g., peer acceptance) have been found to affect the decision to vaccinate [10, 21, 25–27]. Finally, demographic characteristics such as education level have been reported to have less influence than the above-mentioned belief factors on vaccination acceptance [21, 28].

## Methods

This study was conducted using Q-methodology, an approach designed to explore subjective perspectives [29, 30]. Study participants were presented with a sample of opinion statements about HPV vaccination and instructed to rank them according to their agreement with them, thus revealing their subjective viewpoint [29, 30]. Qualitative material was collected by asking participants to explain their statement rankings and answer follow-up questions. Through by-person factor analysis, significant clusters of correlations among rankings were identified. The assumption underlying this analysis was that participants who ranked the statements similarly had similar perspectives on HPV vaccination. For each factor, a composite ranking of the statements was computed, which was the basis for interpretation and description of the factor as perspective on HPV vaccination. As the aim of Q-methodology is to explore the variety of viewpoints that people hold, not to make claims about people expressing them [30], participants were gathered purposively to ensure diversity.

### Statement set development

**Fig. 1:**
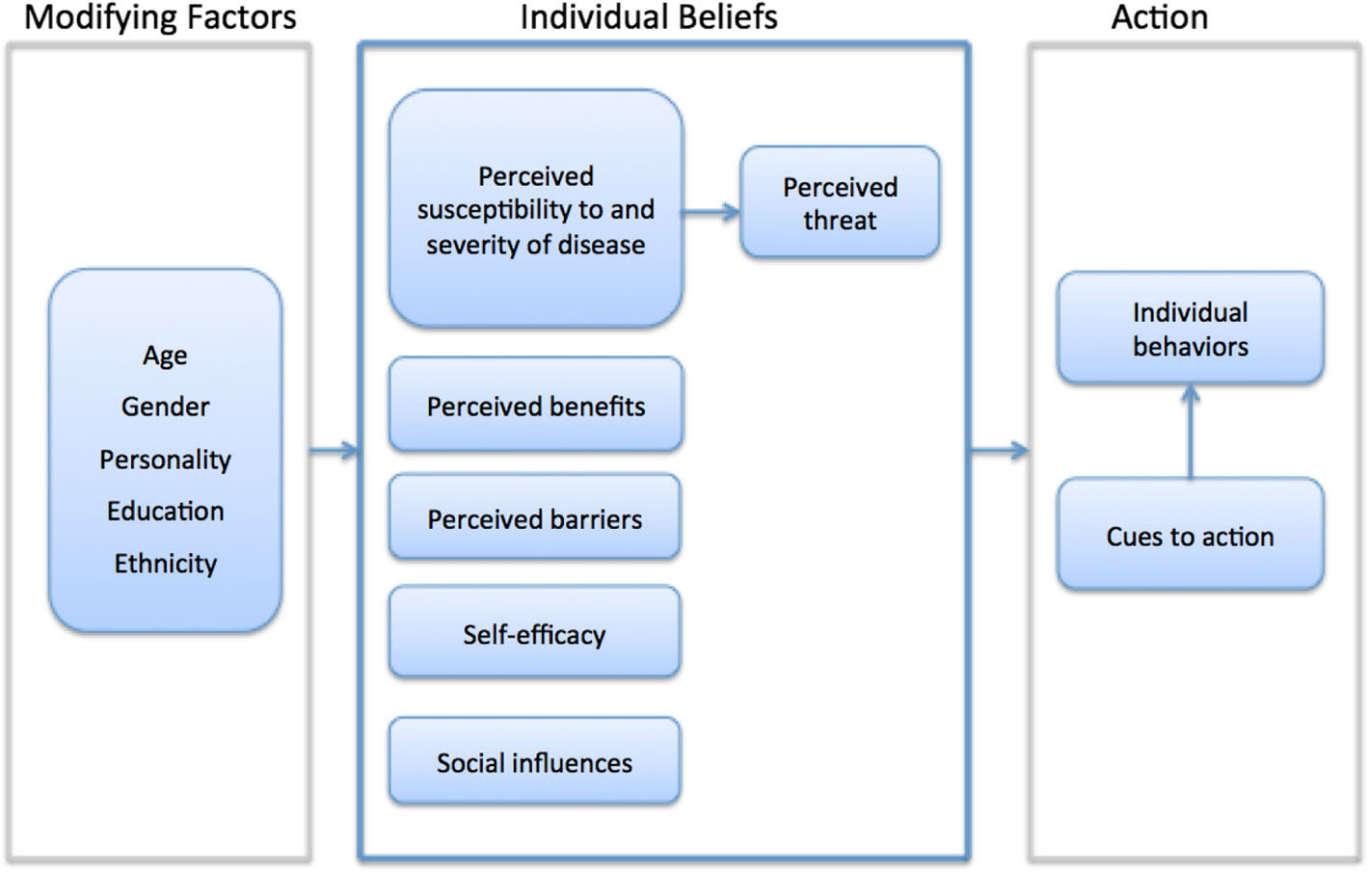
Health Belief Model

To develop a comprehensive and structured set of statements, including all beliefs and motives potentially affecting decisions to vaccinate against HPV, we used the health belief model (HBM) as the theoretical framework for this study. The HBM can help to explain and predict health behaviours [31]. It has proven relevance in various fields of preventive care, including HPV vaccination behaviour [32, 33]. Hence, we used HBM as reference for selecting a set of statements covering the variety of beliefs and motives relevant to our study. The HBM has five major components shaping individuals’ health beliefs: *perceived severity* (seriousness of a disease), *perceived susceptibility* (likelihood of getting a disease), *perceived barriers* (tangible and psychological costs of the action), *perceived benefits* (advantages gained from the action), and *self-efficacy* (belief in one’s ability to execute a behaviour) [35]. Perceived severity and susceptibility have been described as “perceived threat,” which provides the strength and power to act, whereas assessment of the potential benefits and barriers defines the preferred path of action [34]. To accept or follow a preventive action, optimal beliefs about susceptibility and severity are sufficient only when the perceived benefits outweigh the perceived barriers, reducing the perceived threat [34, 35]. Self-efficacy also affects individuals’ health beliefs [34]. In addition to these five major components, an action is further influenced by internal and external “cues to action” (e.g., symptoms, media) and “modifying factors” (e.g., age, education, ethnicity) (34). We added a “social influences” component, involving subjective norms (e.g., beliefs about whether significant others think that one should engage in a behaviour) to the model, based on findings that the social environment and subjective norms (e.g., peer acceptance) are important influences on HPV vaccination decisions [10, 21, 25–27]. Although some researchers consider this factor to be part of the “cues to action” component, others have used it as an additional component in an extended HBM [36, 37]. Figure 1 depicts the adapted version of the HBM used in this study, which is based on Champion and Skinner [34].

Through a non-systematic review of scientific, empirical, and popular literature, two researchers (NP, JC) independently identified a large variety of motives and beliefs potentially influencing decisions to vaccinate against HPV among girls, boys, and parents. The scientific literature was searched using PubMed. In addition, interviews were conducted with two experts in the field of HPV vaccination from two academic medical centres in the Netherlands (one specializing in obstetrics and gynaecology and the other in obstetrics, gynaecology, immunology, and oncology). The two researchers divided the identified motives and beliefs according to the components of the extended HBM, and then reviewed the literature a second time to ensure that nothing had been overlooked.

Four researchers (NP, HD, JE, JC) developed an initial set of 48 statements, and the two experts were asked to evaluate its exhaustiveness. Following their feedback, minor adjustments were made; some statements were merged or deleted because they covered similar topics, and the wording of several statements was revised. Thereafter, two additional HPV policy experts (IW, RB) provided feedback on the statement set, leading to further reduction of the number of statements and additional adjustments to language use. This iterative process resulted in a draft set of 35 statements.

To ensure the applicability and comprehensibility of the statement set, a pilot study involving four parents and six children was conducted. Based on its results, one statement concerning sexual disinhibition (“Vaccination makes you feel protected against HPV, but that is false”) was removed, as it was considered to be outdated. This statement was replaced with statement 11, regarding anticipated regret of inaction, which the pilot study revealed was a missing component. In addition, minor adjustments were made to suit children’s linguistic abilities. The final statement set, categorized according to HBM components, is presented in Table 1.

**Table 1:** Idealized ranking of the 35 statements

Statement		Perspective			
		I	II	III	IV
*Self-efficacy*					
1	You yourself can do something about your health by getting vaccinated against HPV	+3**	-2	+1**	−1
*Cues to action*					
2	I find advice from my GP about HPV vaccination important	+2	0**	+3*	+3
3	Good information can be found about the benefits and drawbacks of HPV vaccination	+1	−2**	−1**	+2
4	Information about HPV vaccination often only deals with the benefits or only with the drawbacks	0	+2	0	+1
5	I am well familiar with HPV and the diseases you can get as a consequence of HPV	0	−1**	−3**	0
6	It is clear to me what HPV vaccination protects against	+1	+1	−4**	+1
7	My decision to get vaccinated depends on the experiences of other people I know	−1**	0	−1	−1
8	I talk with others about the benefits and drawbacks of getting vaccinated against HPV	0	+2**	−2**	0
9	If you can prevent diseases by getting vaccinated, this is a good idea	+4*	+1	+4*	+2
10	I worry about the side effects of HPV vaccination on the long term	0	+4**	+1	0
11	If you don’t get vaccinated and get a disease as a consequence of HPV, you will regret	+2	0	+2	0
12	If you get vaccinated against HPV and get serious side effects, you will regret	0**	+3**	+1	+2
*Perceived susceptibility*					
13	I worry about the diseases you can get if you get infected with HPV	+1*	−1**	+1*	−2**
14	The chance of HPV infection seems small to me	−1**	+1	0*	+1
15	I don't see vaccination against HPV as important	−4**	−1	−2**	−2
16	Vaccination against HPV is only important when you become sexually active	−2	−2	−1	−3**
*Perceived severity*					
17	HPV is not important enough to get vaccinated against	−3**	−1**	−2	−2
18	The diseases you can get due to HPV infection are serious	+3**	+2	0**	+3
*Perceived benefits*					
19	HPV infection is a bigger problem for girls than it is for boys	0	0	0	−1
20	Getting vaccinated against HPV has more drawbacks than benefits	−1**	+1*	0**	0*
21	Getting vaccinated is something you do to protect both yourself and others against infection and diseases as a consequence of HPV	+4**	+1**	+3**	+2**
22	Because you can infect each other with HPV both girls and boys must get themselves vaccinated	+2	0	+2	0
*Perceived barriers*					
23	I am against vaccination in general	−4	−3	−3	−3
24	Deciding to get vaccinated or not is difficult for children	+2**	+3	+2	+1**
25	I have enough information to decide on vaccination yes or no	0**	−1**	−4**	+3**
26	Fear of the needle stick plays a role in the choice of getting vaccinated against HPV	−2**	−4**	−1	−1
27	The possible side effects of HPV vaccination on the long term are not clear to me	+1**	+4**	+4**	0**
28	I find advice from the government about vaccination against HPV important	+3**	0*	+2**	−2**
29	The companies that produce HPV vaccines try to persuade you to get yourself vaccinated	−1	+3**	0	+1**
30	The choice of getting yourself vaccinated against HPV should be made by the parent(s) and child together	+1**	+2**	+3**	+4**
31	A child should decide for herself or himself on getting vaccinated against HPV	−3**	−2**	0**	+4**
32	I find HPV vaccination a tricky topic because it has to do with sexual activity	−2**	−3	−1**	−3
*Social influences*					
33	I would like to know the opinion of others in my close circle (family, friends or classmates) before I decide on getting vaccinated	−1**	0	+1**	−3
34	My decision about HPV vaccination is influenced by my religion or culture	−3	−3	−3	−4**
35	If you get yourself vaccinated against HPV others will think that you are sexually active	−2*	−4	−2*	−4

*p < .05, **p < 0.01 vs. all other factors

### Data collection

An external agency recruited participants purposively to achieve demographic and cultural diversity in terms of age, gender, education level, religion, and ethnicity. In addition, the following inclusion criteria were applied: girls and boys aged 10–16 years who had not yet received HPV vaccination, and parents or legal guardians of such children. Participating parents and children did not have to be from the same families. Initially, three sessions were planned in each of three locations in the Netherlands (Amsterdam, Amersfoort, and Rotterdam). Because 16 participants did not show up, an additional session was organized in Rotterdam. Each session was attended by 6–15 participants. All respondents received €25 gift cards and reimbursement for travel expenses.

**Fig. 2:**
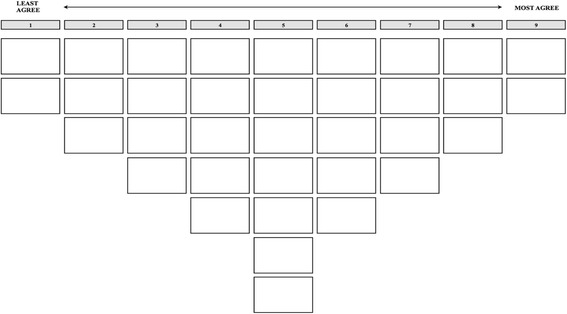
Score Sheet

Each session (moderators: NP, HD) was organized in the following manner: parents and children were separated into different rooms and received a short introduction on HPV (Appendix 1), which provided limited information to minimize potential influence. Participants received written instructions for the exercise. Because these instructions appeared to be too difficult for children in the first session, we switched to verbal instructions for all sessions with children. Thereafter, each respondent was presented with the 35 statements printed on cards, in random order, and asked to carefully read all cards and sort them into three piles representing statements they agreed with, disagreed with, and found to be neutral or irrelevant. The participants were then instructed to reread the cards in each pile, prior to ranking them on the score sheet (Fig. 2). Respondents first placed statements with which they agreed on the right side of the score sheet. They placed the two statements with which they agreed most in the two spots in the far right column, followed by the three remaining statements with which they agreed most, and so on. In the same manner, respondents ranked statements with which they disagreed and those that they found to be neutral on the left side and in the centre of the score sheet, respectively.

After ranking the statements, each participant was asked three open-ended questions. They were asked to elaborate on their choices of the two statements with which they most agreed and disagreed, and to briefly describe their opinion about HPV vaccination. Finally, participants were asked to fill in a questionnaire, which included questions regarding demographic characteristics, knowledge about HPV, HPV vaccination, and their intended vaccination decision.

After data analysis, interviews were conducted with eight participants showing the highest correlations with the identified perspectives (i.e., two participants per perspective). These ex-post interviews were transcribed verbatim.

### Analysis

Individual statement rankings were subject to by-person factor analysis (i.e., centroid factor extraction, followed by varimax rotation) using PQMethod 2.11 [38]. Identified factors were interpreted as perspectives on HPV vaccination using the composite statement rankings. Consensus statements (i.e., those whose rankings did not differ significantly between any pair of factors) and distinguishing statements (i.e., those whose rankings in one factor differed significantly from those in all other factors) were identified. Next, the interpretation and description of each factor was supplemented with the answers to the open-ended questions from participants whose rankings were associated with that perspective (*p* < .05). Finally, ex-post interview data were used to verify and/or clarify interpretations of the factors. Two respondents with the highest factor loadings (i.e. correlations between the respondent and the factors) were selected per factor for the ex-post interviews, as those respondents can be seen as holding the most similar perspective to the one represented by the factor. Two respondents per factor was considered sufficient to verify and clarify interpretations, as no new themes emerged after these interviews.

## Results

The sample consisted of 193 participants [47 (24.4%) girls, 39 (20.2%) boys, 107 parents (55%; 46.1% mothers, 9.3% fathers)]. The average age was 45 (range 29–59) years among parents, and 13 (range 10–16) years among children. Forty-seven parents and children were from the same families (48.7% of the total sample). Four children were excluded from the study because a) they did not complete the exercise, b) understand the instructions after repeated explanation, or c) understand the majority of statements.

Inspection of the solutions supported by the data yielded a four-factor solution. These factors were sufficiently distinct and clearly interpretable, and explained 50% of the variance. Table 1 shows the composite ranking of statements for each perspective.

### Perspective 1: “Prevention is better than cure”

This pro-vaccination perspective, with which 45 participants [35 (78%) parents, 1 (2%) girl, 9 (20%) boys] were associated significantly, emphasized the perceived threat of HPV and HPV-related diseases. An urgency to take preventive measures against HPV prevailed, as reflected in the rankings of statements describing the perceived severity of and susceptibility to HPV and HPV-related diseases. Participants with this perspective believed that they were likely to be exposed to HPV infection (statement 14 was ranked −1), were concerned about its potential consequences to their health (st.13,+1; st.18,+3), and placed importance on prevention through vaccination (st.17,-3).

From this perspective, the benefits of HPV vaccination clearly outweighed potential barriers. Vaccination was considered to be a positive means of preventive care to protect oneself and others (st.21,+4), with more advantages than disadvantages (st.20,-1), giving people the direct ability to influence their health (st.1,+3). Taking preventive measures was seen as a small sacrifice relative to the potential consequences of HPV-related diseases, as highlighted in several answers to open-ended questions with the recurring idiom of “prevention is better than cure.”

Another theme that emerged within this viewpoint was the perceived complexity of HPV and HPV-related diseases, reflected in beliefs about *who* should make the vaccination decision [i.e., not children alone (st.31,-3) or parents and children together (st.30,+1)]. Parents stated that they would rather announce this decision to their children than negotiate it with them. They felt strongly that children receiving formal invitation letters for local vaccination sessions could not understand such complex information and were incapable of making informed decisions.

Given the perceived complexity of the topic, participants with this perspective valued professional advice from trusted sources, such as governmental recommendations (st.28,+3). This perception may explain the minimal value placed on social influences (st.33,-1; st.34,-3; st.8,0): one mother said, “I do not rely on the opinion of my neighbor or sister-in-law; I collect good information from the Internet about pros and cons and then make my choice.” These participants’ trusting relationships with the government may additionally explain their generally favorable opinion of HPV vaccination. In ex-post interviews, respondents opined that the government would not add a vaccine to the NIP without proper and relevant justification. In the context of healthcare and the “medical world” in the Netherlands, participants with this perspective trusted the government to make good, wise choices with people’s best interests in mind.

### Perspective 2: “Fear of unknown side effects”

This perspective, with which 46 participants [40 (87%) parents, 4 (9%) girls, 2 (4%) boys] were associated significantly, did not involve the strength and force to take action against HPV through vaccination, as the perceived severity and susceptibility of HPV-related disease was minimal. The perceived likelihood of exposure to HPV-related disease was minimal (st.14,+1) and concern about contracting HPV-related disease was negligible (st.13,-1). Hence, respondents with this perspective did not perceive HPV to be sufficiently important to warrant vaccination (st.17,-1).

Perceived barriers, particularly fear about the potential unknown long-term side effects of HPV vaccination (st.10,+4; st.27,-4), dominated this perspective. In this way, HPV vaccination was distinct from other childhood vaccinations (st.23,-3). One mother stated, “[I have] concerns about the side effects in the long term. The vaccine was developed only recently. The possible long-term side effects cannot be known yet.” Respondents also referred to the effectiveness of the vaccine, emphasizing that the current vaccine does not protect against all cancer-causing HPV strains. One interviewee also questioned the duration of the vaccine’s protection against HPV infection.

Respondents’ fears were also linked to mistrust of pharmaceutical companies, perceived as profit-making entities that wanted to sell their products, even at the cost of others’ well-being (st.29,+3). Participating mothers stated, “Ah yes, surely they make plenty of money with it. Without really telling the truth about the severe side effects” and “This is nothing new. The pharmaceutical companies often are big crooks with dollar signs in their eyes. The humane element in the pharmaceutical industry has gone, regrettably.” This mistrust of the pharmaceutical industry and the prevailing fear about potential negative consequences made respondents perceive children vaccinated against HPV as test objects; one mother said, “I don’t really want my daughter to act as a guinea pig or for people to make money out of her at the cost of her health.” Thus, these participants considered it better to be safe and not get vaccinated, than to regret vaccination due to potential future side effects (st.12,+3). However, they also expressed that only the future could tell whether they had made the right decision.

This perspective also involved a lack of trust in the government to give advice about HPV vaccination (st.28,0). One interviewee referred to the government’s hasty choice to include the HPV vaccine in the NIP. In addition, these respondents placed little value on the role of GPs relative to those with other perspectives (st.2,0).

Participants with this perspective perceived that adequate and objective information on HPV vaccination was difficult to find (st.3,0; st.4,+2), which may partly explain the perception that they were inadequately informed about HPV and related diseases (st.5,-1). In addition, they perceived HPV as a complex topic and thus felt that parents should make vaccination decisions (st.24,+3). One respondent emphasized that her daughter was very compliant, with a constantly altering opinion depending on with whom she had spoken.

### Perspective 3: “Lack of information and awareness”

The defining feature of this perspective, with which 38 participants [14 (37%) parents, 14 (37%) girls, 10 (26%) boys] were associated significantly, was lack of awareness and information about HPV and HPV-related disease, which posed a major barrier to informed decision making (st.25,-4; st.6,-4). Many participants stated that the topic of HPV was completely unclear for them, and some had not heard about HPV before this study. Interviewees linked HPV with cervical cancer, albeit diffusely. Given their limited awareness and information, they pondered the advantages and disadvantages of vaccination, being unclear about its potential side effects (st.27,+4) while perceiving the benefits of vaccination in general, and HPV vaccination specifically, as important (st.9,+4; st.21,+3).

This perspective involved the least perceived severity among viewpoints of the potential consequences of HPV-related disease (st.18,0) and a neutral perception of susceptibility (st.14,0; st.13,+1). Yet, interviewees stated that cervical cancer could be severe. This contradictory view could be explained by the expressed lack of information, awareness, and clarity regarding the link between HPV and (cervical) cancer.

Respondents valued friends’ and family members’ opinions about the HPV vaccine (st.33,+1), and thus appeared to be influenced more easily by the social environment. In contrast to participants with other perspectives, those with this viewpoint valued GPs’ advice highly (st.2,+3). A 14-year-old girl said, “I think advice from the GP is important because you don’t know the drawbacks of an HPV shot. There is not enough information about the HPV shot” and a father stated, “Explanation by a GP gives you some more certainty! He is a specialist after all.”

Interviewees wished that they knew more about HPV and HPV-related disease. When asked how information should be distributed, one respondent suggested more active communication about the vaccine through spot advertisements and increasing awareness through GPs and informational pamphlets distributed with formal invitations for other vaccinations.

In addition, many children holding this view appeared to trust and rely on their parents’ decision, but felt that they should have a say in this decision despite their limited knowledge about the HPV vaccine. A 13-year-old girl, who had declined vaccination, stated that she would have liked to know more about HPV before making a decision and expressed anticipated regret if she were to acquire an HPV-related disease later in life. However, she declined having plans to rethink her decision.

### Perspective 4: “My body, my choice”

The 22 participants [2 (9%) parents, 15 (68%) girls, 5 (23%) boys], in large majority girls, associated significantly with this perspective did not perceive HPV-related diseases as a particular threat to health. Perceived susceptibility was especially low, as respondents did not seem to worry about exposure to HPV and related diseases compared with those with other perspectives (st.13,−2). One 14-year-old girl stated that she had decided to decline the HPV vaccine, but not other childhood vaccinations: “…these I felt were important and therefore I accepted them.” This viewpoint was further justified by the fact that common childhood vaccinations prevent diseases perceived as more common than HPV-related disease.

Yet, respondents felt clearly that making the decision to vaccinate before becoming sexually active was important (st.16,-3). Although they believed that vaccination in general benefitted oneself and others (st.21,+2), respondents representing this perspective seemed to be undecided about whether HPV vaccination has more disadvantages than advantages (st.20,0). One interviewee referred to unknown side effects, and another referred to the effectiveness of the vaccine, stating she had heard that the vaccine’s ability to protect against cervical cancer was uncertain.

The most important aspect of this viewpoint was control over the decision of whether to vaccinate. Children believed that they should make this decision (st.31,+4), positioning themselves (as vaccine recipients) centrally in responses to open-ended questions and in ex-post interviews; a 15-year-old girl stated, “It’s your body so I think you may take the decision yourself.” Children also expressed that their opinions were equally important as their parents’, if not more important: 14- and 13-year-old girls stated, “Well, they thought my opinion was really important; so I didn’t have to get it if I didn’t want it” and “Yes, [my parents] thought my opinion was more important.”

However, children also valued their parents’ knowledge and saw the decision as joint (st.30,+4): 12- and 14-year-old girls said, “But it may be that as a child you are not too sure about what it involves, so therefore I find it important that parents also have a say in this” and “As a child you don’t know much about this but your parents just a bit more, so it’s useful to have them help decide.”

A probable cause for this rather contradictory view on who should make the decision is that children want to be able to decide who can influence the decision: preferably only people from their “circle of trust” (st.33,-3). Yet, those with this perspective greatly appreciated GPs’ recommendations (st.2,+3); a 13-year-old girl said of her GP, “He knows me,” and referred to GPs’ medical knowledge about their clients. Yet, neither girl with this perspective who was interviewed had actively reached out to her GP for advice, despite having decided to decline the vaccination. Expert advice from other sources, such as the government, was not considered to be relevant (st.28,-2). However, most children did not appear to fully understand the term “government” or what it entailed*.*

Lack of information did not appear to be a barrier for respondents with this perspective (st.25,+3), even those who expressed that they knew little about HPV and HPV-related disease. Compared with participants with other perspectives, they felt that knowledge, when needed, was easily obtainable from parents or GPs (st.3,+2).

## Discussion

Although many studies have explored beliefs regarding the HPV vaccine, this study is the first to investigate jointly the prevailing perspectives of girls, boys, and parents, and to identify determinants underlying HPV vaccination decisions. The results revealed four perspectives: “prevention is better than cure,” “fear of unknown side effects,” “lack of information and awareness,” and “my body, my choice.”

Our results suggest that the first two perspectives were coherent and very distinct. Those with perspective 1 favoured vaccination, as it is not only promoted by a trusted source (the government), but is also an effective means of preventing HPV-related disease, thereby minimizing perceived susceptibility and severity. These determinants coincide strongly with previous findings about HPV vaccine uptake [9, 27, 33]. In contrast, those with perspective 2 did not view HPV as a particular threat to health and were critical of the roles of the government and the pharmaceutical industry in HPV vaccination. Their inclination to not vaccinate was dominated by fear of potential long-term side effects, in accordance with findings about HPV vaccination refusal [9, 10, 14, 15]. In relation to the HBM, those with perspective 1 are highly likely to accept HPV vaccination, as the perceived threat is seen as considerable, perceived benefits outweigh barriers, and self-efficacy is present. Furthermore, some cues to action can also be seen as additional “triggers” positively influencing uptake. On the other hand, those with perspective 2 are unlikely to accept HPV vaccination, as the perceived threat is minimal and the perceived barriers clearly outweigh any benefit. Furthermore, cues to action indicate a need for more objective and adequate information. However, it is unclear *what* information would be adequate and by *whom* – other than government or the pharmaceutical industry – it should be provided to satisfy them. In both of these perspectives, the vaccination decision seems to be well considered and clear.

In contrast, the determinants underlying perspectives 3 and 4 appear to be ambiguous and, to some extent, incoherent. Those with perspective 3 clearly showed a lack of awareness and information needed to make informed decisions about HPV vaccination. This perspective appears to involve doubt about vaccination, as contracting HPV infection or HPV-related disease was not perceived as a particular threat, and neither benefits nor barriers clearly carried more weight. Previous studies have shown that knowledge has different effects on vaccination decisions. Lack of knowledge or information can be a reason to decline vaccination [18], but it is not necessarily a factor in vaccination rejection [14, 26]. Those with this perspective appeared to be more easily influenced by their surroundings, valuing the opinions of others and their GP; children tended to rely on their parents’ vaccination decision. In contrast, and despite potential influences from social surroundings, those with perspective 4 wanted children, who ultimately experience the consequences, to actively control final vaccination decisions. These respondents also felt that they possessed sufficient information to make vaccination decisions, although study data suggest that they knew very little about HPV (see discussion paragraph 6 and 7).

In relation to the HBM, the vaccination decision in perspective 3 can be interpreted as being in a modifiable phase, given the clear lack of vaccination preference and prevailing emotions regarding the topic. Those with this perspective are not likely to vaccinate against HPV, as they feel no sense of urgency in terms of perceived threat or benefit. Contrary to those with the first two perspectives, the deliberations of those with perspective 3 appear, at best, to be characterized by doubt. Those with perspective 4 (largely children) are arguably also in a modifiable phase, with minimal awareness and perceived severity and susceptibility of HPV and HPV-related disease, and no clear idea of HPV immunization benefits and barriers. The combined lack of awareness and focus on children’s roles in vaccination decision suggest that these individuals are overconfident in children’s ability and willingness to acquire and weigh the required information to make well-considered decisions about HPV vaccination.

Interestingly, and significant for the future uptake of this new generation of vaccines of which HPV is considered to be the first, the importance of individual deliberation and choice in immunization programs and vaccination policies is increasing [39]. In many countries, including the Netherlands, childhood vaccination is portrayed as “the natural thing to do” and considered to be part of “responsible parenting” [40]. As the study results indicate, attitudes about HPV vaccination deviate from this institutionalized “immunization logic”; this vaccination is seen as a choice for which potential users bear private responsibility. This situation poses a new policy challenge for governments and public health authorities to inform and potentially “persuade” target groups to participate in vaccination programs, thereby rendering them effective. Individuals facing the choice of whether to vaccinate against HPV need tailor-made information, which must address subgroups with diverse beliefs and health orientations. Furthermore, this new perception of choice may spill over to other vaccinations included in the NIP, potentially leading to (re)emphasis of their possible side effects and dangers. This was the case in the 1990s, when measles, mumps, and rubella vaccination was said to cause autism, leading to a decline in its uptake [41, 42]. This shifting vaccination logic requires the rethinking of immunization policies [39].

Although the participants in this study were sampled purposively, exploring associations between perspectives and background characteristics may generate interesting ideas for further research. Firstly, most parents held perspectives 1 and 2. Most children held perspectives 3 and 4, or were not associated with any perspective, possibly indicating lack of clear perspective about this topic. Secondly, perspectives were not associated with parents’ gender, but more girls were associated with perspective 4 and more boys with perspective 1. Thirdly, perspectives were not associated with parents’ education level, but children with lower education more often associated with perspectives 1, 4, or had no clear perspective. Fourthly, participants with perspectives 1 and 2 scored higher on personality trait conscientiousness, while those with perspective 4 scored lower; other Big Five Inventory [43] personality traits showed no associations.

The questionnaire also included questions about HPV vaccination. Firstly, parents who had received the formal invitation letter for their child’s vaccination more often held perspectives 2 and 4, whereas more of those who had not yet received this letter associated with perspectives 1 and 3, or no perspective. Secondly, those with perspective 2 more often had actively searched for information about HPV vaccination, and those with perspective 3 or no clear perspective less often. Thirdly, those with perspective 2 scored higher on a knowledge of HPV scale, adapted from Gefenaite et al. [15]. This coincides with earlier findings that those critical of new technologies are often also highly informed [44]. Finally, more parents who had discussed about the HPV vaccine with others held perspective 2, whereas more children who had talked about the HPV vaccine with others held perspective 4.

The findings of this study are important for several reasons. First, the modifiable-phase status of those with perspectives 3 and 4, who have not (yet) made well-considered decisions about HPV immunization, emphasizes the need for investment in tailor-made independent communication policies (e.g., spot advertisements, informational pamphlets accompanying invitations to preceding vaccine sessions, promotion of contact with GP or other medical professionals for advice) to enhance HPV immunization. A focus on increasing awareness and knowledge is particularly important among those with perspectives 3 and 4, who appeared to be rather unfamiliar with the topic of HPV. The involvement of adolescents in decision-making is a particularly important opportunity to provide HPV education. HPV awareness could be increased by incorporating informational sessions in schools’ sexual health education classes. When providing information to those with perspectives 3 and 4, a focus on solidifying the link between HPV and cervical cancer, and emphasis that cervical cancer is only one of many severe consequences of persistent HPV, would be beneficial. Second, as suggested by Griffioen et al. [25], there appears to be a missed opportunity in educating children of parents with perspective 1, who make HPV vaccination decisions for their child without discussion given the perceived complexity of the topic. Parents should be encouraged to have open dialogs with their children, which clearly differs from perspective 4. However, HPV vaccination decision making may become tense when parents and children hold different perspectives.

This study has some limitations. First, as the aim of a Q-methodological study is to describe prevailing perspectives, the current data do not indicate how common these perspectives are in society; such information, acquired with a survey approach [45], could be valuable for assessing whether and how vaccination rates can be further improved. The same applies to relations between respondents’ perspectives and background characteristics. The associations discussed above are tentative and, although some have clear face validity, should be seen primarily as hypotheses for further research. One of the reasons underlying the different perspectives among girls and boys may be that HPV vaccination is part of the NIP only for girls in the Netherlands; thus, boys and their parents do not receive a formal invitation letter, which may influence their deliberation, awareness and perspective on HPV vaccination. Second, a non-systematic literature review was conducted to develop the set of statement, which does not guarantee elimination of bias. The process, however, of developing the statement set involved two researchers independently identifying a large variety of motives and beliefs potentially influencing decisions to vaccinate against HPV, twice the input from two experts, and a pilot among children and parents to ensure that nothing had been omitted. Third, although much emphasis was placed on the development of a statement set that would be suitable for parents and children, some adjustments had to be made after the pilot study to fit children’s linguistic abilities and varying levels of HPV awareness. During the main study, no further concerns arose about the comprehensibility and completeness of the statements except that the term “government” needed clarification for some children. Finally, statement categorization within the HBM was challenging, especially as the relationships among HBM components are not clearly defined [34] and clear construct definitions are lacking, particularly for the cues to action component [31]. Therefore, some statements could have been classified differently; in particular, some of those categorized as social influence could also have been classified as cues to action. However, we believe that the HBM functioned as a comprehensive framework for developing the statement set, as confirmed by the fact that no main study participant reported missing an aspect important to their perspective.

## Conclusions

Our study identified four prevailing perspectives on HPV vaccination among girls, boys and parents. Given the aim of publically funded vaccination programs to minimize the spread of HPV infection and HPV-related disease and concerns about current uptake, knowledge of existing perspectives on HPV vaccination in the public is crucial for policies wishing to promote uptake. HPV vaccine clearly deviates from other childhood vaccinations in NIP’s, as vaccinating against HPV is not necessarily considered “the natural thing to do”. Increasing awareness and knowledge by investing in tailored-made communication policies could help promote the uptake among those not familiar with the vaccine. This is especially important among those holding perspective 3 or 4, who appeared to be in a modifiable phase. Despite the non-generalizability of this study, the methods and results of this study could contribute to the development of campaigns wishing to address uptake of HPV vaccination.

## Appendix 1

We conduct this study because we are curious to know what children and parents think about HPV vaccination. HPV stands for human papillomavirus. There are different variants of this virus, with different effects on your health. HPV infection can occur in two ways: through skin-to-skin contact or via sexual contact. The variants of HPV that can lead to severe diseases are transmitted through sexual contact.

## Abbreviations

HBM: Health belief model
HPV: Human papillomavirus
NIP: National immunization program
